# Positive predictive value of the German notification system for infectious diseases: Surveillance data from eight local health departments, Berlin, 2012

**DOI:** 10.1371/journal.pone.0212908

**Published:** 2019-02-22

**Authors:** Benjamin Blümel, Michaela Diercke, Daniel Sagebiel, Andreas Gilsdorf

**Affiliations:** 1 Postgraduate Training for Applied Epidemiology (PAE, German FETP), Department of Infectious Disease Epidemiology, Robert Koch Institute, Berlin, Germany; 2 European Programme for Intervention Epidemiology Training (EPIET), European Centre for Disease Prevention and Control (ECDC), Stockholm, Sweden; 3 Department of Infectious Disease Epidemiology, Robert Koch Institute, Berlin, Germany; 4 Berlin State Office for Health and Social Affairs, Berlin, Germany; University of Zurich, SWITZERLAND

## Abstract

The German Infection Protection Act requires notifying certain cases of infectious diseases to local health departments (LHD) in Germany. LHDs transmit notifications meeting case definitions to the national health authority, where the proportion of discarded notifications is not known. The proportion of discarded cases at the level of LHDs can be expressed as the positive predictive value (PPV) of the notification system. The PPV can be used to assess the efficiency of the system. We quantified the proportion of discarded notifications to calculate the PPV of the German notification system at the level of LHDs using electronic notification data from Berlin LHDs from 2012. We also analysed reasons for discarding notifications by reviewing notification forms. Data was available from eight LHDs (67%) receiving 10,113 notifications in 2012. Overall PPV was 89% (minimum-maximum = 77–97% across LHDs) and ranging from 30% (Hepatitis B) to 99% (Rotavirus). Of 166 individual investigation forms 84% were on hepatitis B or C cases, most of them discarded because of previously diagnosed chronic disease. LHDs investigate many notifications that do not lead to public health action and useful surveillance data leading to inefficient use of resources. Adaptation of case definitions or the legal framework concerning notifications may increase the efficiency of the notification system and lead to better use of data from notified cases.

## Introduction

Regular evaluation of a surveillance system ensures its efficiency and effectiveness [1]. National and international guidelines for the evaluation of surveillance systems recommend the use of surveillance system attributes such as simplicity, flexibility, data quality, acceptability, sensitivity, positive predictive value (PPV), representativeness, timeliness, and stability to identify gaps and to further develop and improve the system [2–7].

Since 2001 many studies evaluated attributes of the German surveillance system including simplicity, acceptability, data quality, PPV, and timeliness. The studies examined system attributes at different levels of the surveillance system (local, regional, and national) either in a disease-specific or a more general, system-wide way [8–13]. Only one study examined the PPV of the German notification system from the viewpoint of the regional and national levels not including data from the local level [11].

The PPV in the context of a surveillance system is the proportion of all notifications that, after validation, are defined as true cases according to the case definition of the surveillance system [2]. A low PPV means that many notifications are not actual cases (according to the surveillance system case definition), false positive notifications are common. These notifications may still have to be investigated and if the proportion of false positives is very high, public health resources might not be used efficiently [2]. Therefore, the PPV is very important in the context of the limited resources available to the public health system and may be used to describe the efficiency of the system.

**Fig 1:**
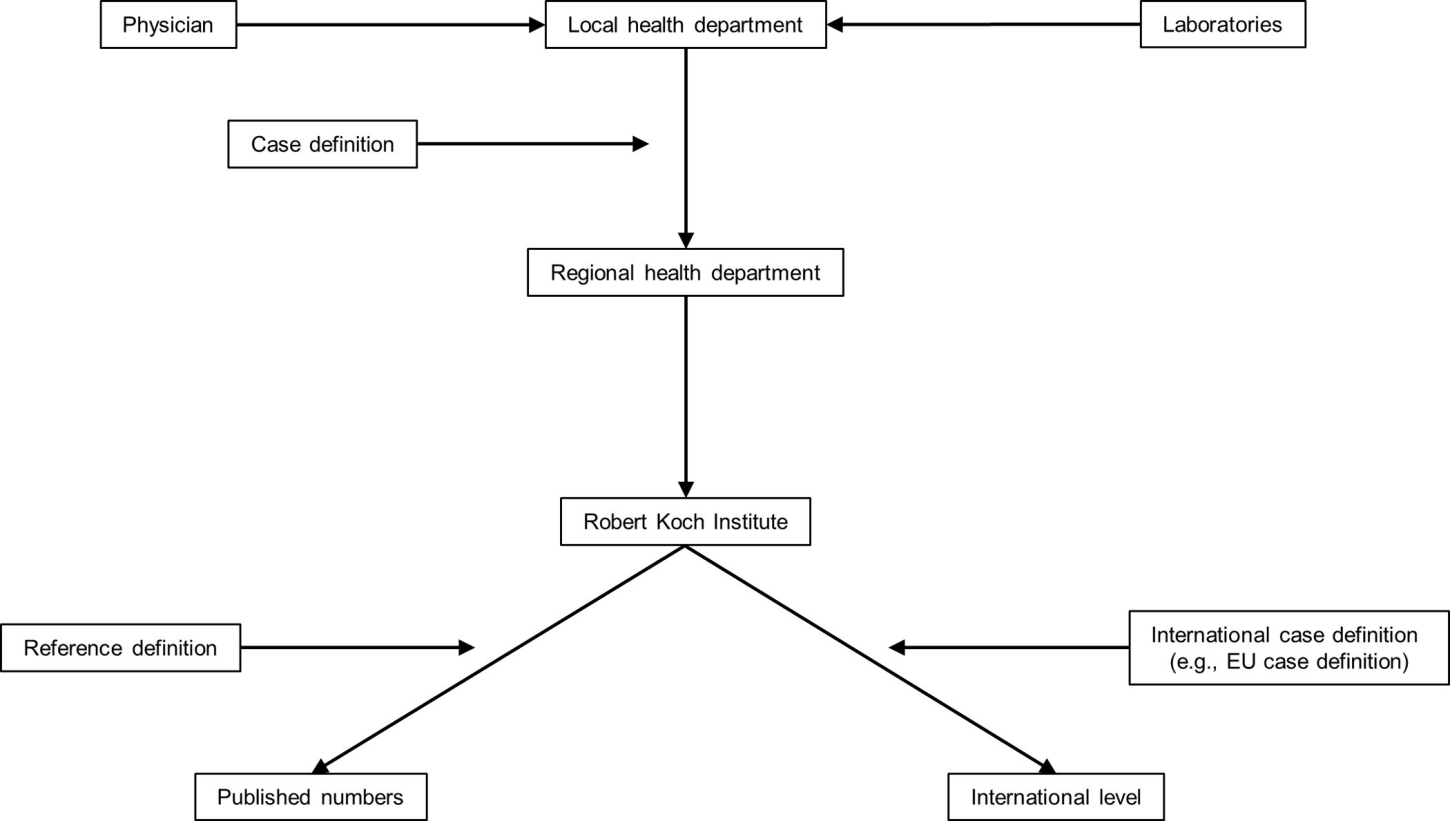
Flowchart of the German notification system for infectious diseases.

Since 2001, surveillance of infectious diseases in Germany is regulated by the Infection Protection Act (Infektionsschutzgesetz) [14]. According to this law, physicians and laboratories are required to notify certain infectious diseases (suspected cases, illness or deaths from these diseases) or the detection of certain pathogens to local health departments (LHDs). The investigation of notifications and the implementation of measures is the responsibility of LHDs. After investigation, LHDs transmit notifications that meet the German case definitions electronically and anonymised to the regional health departments. These then transmit cases further to the national health authority (Robert Koch Institute, RKI) that monitors trends in case numbers and conducts epidemiological analyses. Cases are filtered further by so called reference definitions. Final case numbers are then published by the Robert Koch Institute in weekly and yearly reports and on its website (www.rki.de/survstat). Based on respective case definitions case-based data is transmitted further to the international level (Fig 1).

German case definitions are developed and published by the Robert Koch Institute [15]. The main purpose of case definitions is the systematic validation and standardized assessment of notifications across Germany. Their use contributes to increased comparability of data across states and thus to improved surveillance data quality resulting in more reliable epidemiological analyses and recommendations for public health action. They are only used by health authorities, mainly at LHDs, and do not define criteria for notification or clinical diagnosis. Reference definitions are used to define which cases are considered true confirmed cases. These case numbers are then published in official RKI publications.

As LHDs are not required to transmit notifications that do not meet case definitions, the total number of notifications and thus the proportion of transmitted cases among all notifications (i.e., the PPV of the notification system) are not known at the national level. Consequently, the reasons for discarding notifications and the associated workload are also not known at the national level. In this study we aimed to quantify the PPV of infectious disease notifications in Germany to assess the efficiency of the notification system. We also aimed to identify reasons for discarding notifications and the diseases causing the most difficulties at LHDs in this concern.

## Methods

### Estimating the PPV

We conducted the study in the city state of Berlin because there the same software is used in all LHDs for the management of infectious disease notifications. Additionally, in Berlin regularly all notifications are entered in the electronic system as the number of notifications is used to estimate the human resources needed for infectious disease surveillance within the LHD. We collected data in 2013 for the year 2012 as this was the most recent data available.

We provided all LHDs in Berlin (n = 12) with detailed instructions explaining how to extract data stratified by disease, the number of notifications received at the LHD for all diseases that are notifiable to LHDs according to IfSG. For hepatitis C, we asked LHDs to extract data also by age group (0–24, 25–44, 45–64 and above 64 years) to evaluate differences of the PPV of a disease with increasing prevalence over age groups [16]. Tuberculosis was not included in our study as the data is collected by one specialised LHD for the whole city state. At RKI, we extracted by disease the number of notifications received at the national level from Berlin in 2012 and among them the number of those that meet the reference definitions.

**Fig 2:**
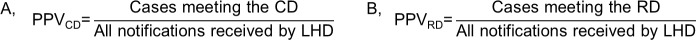
**Formulae of the positive predictive value based on the case definition (PPV**_**CD**_**, A) and the reference definition (PPV**_**RD**_**, B).** CD: case definition; RD: reference definition; LHD: local health department.

We defined the proportion of cases that meet the case definition and are transmitted by LHDs to the regional level and RKI among all notifications received at the LHD as the PPV based on the case definition (PPV_CD_, Fig 2A). Additionally, we calculated the proportion of cases that meet the reference definition among those received at the LHD (PPV based on the reference definition (PPV_RD_, Fig 2B). PPVs were calculated overall and by disease. For hepatitis C we calculated the PPV_CD_ by age group. For each PPV the highest and lowest values were provided (minimum-maximum (min-max)) across LHDs. Disease-specific PPVs were calculated for diseases with at least 10 notifications and was not calculated for suspected rabies exposure. Diseases with less than 10 notifications and suspected rabies exposure are shown in the category “Other” with PPV_CD_ and PPV_RD_ without minimum or maximum values. The definition of PPV used in this study does not aim to represent information about the certainty of the clinical diagnosis. The PPV shows what proportion of all notifications are considered to be true cases for surveillance purposes and should be interpreted as such [3].

### Reasons for discarding notifications

In one LHD we reviewed individual investigation forms of discarded notifications available as paper forms from the archive to determine whether the notifications had been investigated. If they had been investigated, we analysed whether further measures for case management by the LHD were necessary. We considered an investigation having taken place when the patient, the treating physician or the laboratory had been contacted and thus at least one item of the first part of the investigation form (detailed demographic data, data about symptoms and history of the disease, medical history of the patient, laboratory data) was filled out. We considered measures having taken place when the “measures” part of the investigation form had been filled out. Measures included, among others, informing the patient, family members or contact persons about the disease; recommendation for vaccination; contact tracing. We collected only anonymised data and all results are presented as aggregated data.

All analyses were carried out with Microsoft Excel 2010 and STATA version 12.1 (StataCorp, College Station, TX, US).

No ethical approval was needed for this study as only anonymised surveillance data was used for the analyses.

## Results

### Estimating the PPV

**Fig 3:**
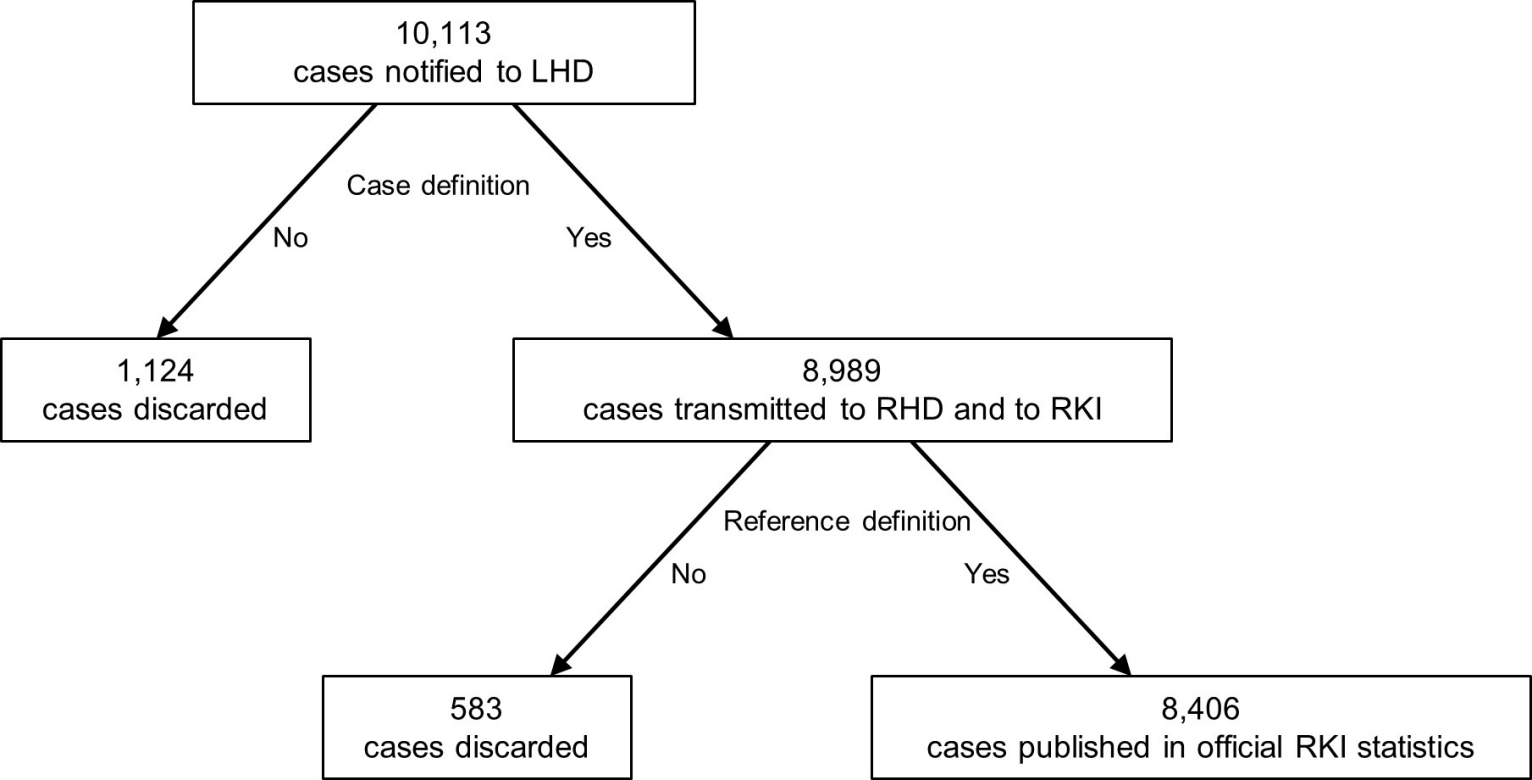
Notified and discarded infectious disease notifications, Berlin, 2012. LHD: local health department; RHD: regional health department; RKI: Robert Koch Institute.

Nine (75%) of the twelve LHDs in Berlin extracted and provided data for the study. One LHD was excluded as it declared that it did not keep all notifications in the electronic system. Hence, data from 8 LHDs (67%) could be included in the analyses. Overall, in 8 LHDs, 10,113 notifications were recorded in 2012. 8,989 notifications were transmitted resulting in an overall PPV_CD_ of 89% (min-max: 76–97% across LHDs). 8,406 cases met the reference definition resulting in an overall PPV_RD_ of 83% (min-max: 73–89% across LHDs) (Fig 3). PPV_CD_ was lowest for hepatitis B (PPV_CD_ = 30%, PPV_RD_ = 10%), hepatitis C (PPV_CD_ = 43%, PPV_RD_ = 43%) and meningococcal meningitis (PPV_CD_ = 55%, PPV_RD_ = 55%) (Table 1). Across diseases, PPV_CD_ and PPV_RD_ was highest for rotavirus gastroenteritis (99%, min-max: 98–100%; 98%, min-max: 97–99% respectively).

**Table 1:** Positive predictive value of infectious disease notifications based on the case definition and the reference definition, number of notifications received, number of notifications meeting the case definitions and reference definitions, Berlin, Germany, 2012.

Notification category	PPV_CD_ (%)	Min-max (%)	PPV_RD_ (%)	Min-max (%)	N received	N meeting CD	N meeting RD
Hepatitis B	30	9–65	10	0–16	480	142	47
Hepatitis C	43	21–65	43	21–65	960	409	408
Meningococcal meningitis	55	20–100	55	20–100	31	17	17
Measles	56	0–100	56	0–100	27	15	15
Invasive MRSA^a^ infection*	85	40–100	85	40–100	249	212	212
Hepatitis E	88	0–100	76	0–100	17	15	13
Invasive *H*. *influenzae*^b^ infection*	88	50–100	88	50–100	16	14	14
Hepatitis A	89	74–100	77	50–92	79	70	61
Adenovirus conjuctivitis*	91	60–100	86	60–100	43	39	37
Listeriosis*	93	75–100	80	50–100	15	14	12
Legionellosis*	95	80–100	84	50–100	43	41	36
VTEC^c^ infection*	96	83–100	82	50–100	51	49	42
Yersiniosis*	97	83–100	87	57–100	60	58	52
Salmonellosis*	97	87–100	89	86–98	508	492	454
Influenza*	98	75–100	97	75–100	291	285	282
Non-VTEC *Escherichia coli* enteritis*	98	94–100	86	78–100	369	361	316
Dengue fever*	98	94–100	93	83–100	42	41	39
Shigellosis*	99	93–100	97	86–100	78	77	76
Norovirus gastroenteritis*	99	94–100	89	84–95	2,668	2,629	2,372
Cryptosporidiosis*	99	96–100	93	75–100	105	104	98
Campylobacteriosis*	99	97–100	97	95–99	2,306	2,273	2,229
Giardiasis*	99	97–100	90	75–100	384	380	345
Rotavirus gastroenteritis*	99	98–100	98	97–99	1,234	1,224	1,206
Other	49	-	40	-	57	28	23
All	89	76–97	83	73–89	10,113	8,989	8,406

PPV, positive predictive value; min-max, minimum-maximum; CD, case definition; RD, reference definition.

^a^ Methicillin-resistant *Staphylococcus aureus*.

^b^ Haemophilus influenzae.

^c^ Verotoxin-producing *Escherichia coli*.

*Notifiable only by the laboratory.

Reference date for data extraction: 01.08.2013.

Diseases that are notifiable by physicians and laboratories (e.g. meningococcal meningitis, measles) had a lower overall PPV compared to those notifiable only by laboratories (PPV_CD_ = 42%, min-max: 28–69% versus PPV_CD_ = 98%, min-max: 92–100%; PPV_RD_ = 35%, min-max: 22–54% versus PPV_RD_ = 92%, min-max: 89–95%).

Hepatitis C data for age group specific PVP_CD_ was available from 7 LHDs. PVP_CD_ was higher in the younger age groups and decreased with age (0–24 years: 61%, min-max: 0–100%; 25–44 years: 50%, min-max: 26–68%; 45–64 years: 40%, min-max: 20–70%; above 64 years: 34%, min-max: 0–60%).

### Reasons for discarding notifications

Of 166 investigation forms of discarded notifications available from one LHD, 57% indicated that the LHD had conducted investigations. For 24% of the investigated notifications (14% of all 166 discarded notifications) further measures were necessary. Most discarded notifications were either for hepatitis B (n = 47) or hepatitis C (n = 93). Other diseases were measles, meningococcal meningitis, tick-borne encephalitis, invasive infections with methicillin-resistant *Staphylococcus aureus* (MRSA), rotavirus gastroenteritis, norovirus gastroenteritis, giardiasis, campylobacteriosis, salmonellosis, and cryptosporidiosis. The most common reason for discarding hepatitis B and C notifications was chronic hepatitis that was either previously diagnosed or notified (the patient or the physician reported the disease to be known or the LHD found information about the person in its database) or for hepatitis B newly diagnosed chronic disease. Other reasons were that the notified person could not be identified or reached, was a foreigner, or did not have the clinical symptoms necessary for the case definition to be met. For diseases other than hepatitis B or C, the most common reasons for discarding the notification were that the person could not be identified or reached, was a foreigner, did not have clinical symptoms necessary for the case definition to be met, that a specimen other than the one required by the case definition was taken, or that a laboratory method other than the one required by the case definition was used (Table 2).

**Table 2:** Reasons for discarding notifications of hepatitis B, C and other infectious diseases at one local health department, Berlin, Germany, 2012 (n = 166).

Reason for discarding	Hepatitis B (n = 47)	Hepatitis C (n = 93)	Other (n = 26)
Chronic disease, previously diagnosed or notified	72%	83%	0%
Chronic disease, newly diagnosed	11%	1%	0%
Person could not be identified or reached or foreigner	12%	17%	50%
Clinical symptoms necessary for CD missing	4%	0%	8%
Specimen or laboratory method other than one required by CD	0%	0%	12%
Other	0%	1%	31%

CD: case definition. Overall percentages may not equal to 100% because of rounding.

## Discussion

### PPV

In this study we quantified the proportion of discarded notifications in the German notification system at the level of LHDs using data from the city state of Berlin. Considering all diseases, around 11% of notified cases are discarded by LHDs in Berlin. Extrapolating this percentage on the number of notifications received at the regional and national levels (around 300,000 notifications per year, data source: www.rki.de/survstat) we can assume that German LHDs discard around 33,000 of them. There is a wide range of these proportions between diseases with more than two thirds of hepatitis B and only 1% of rotavirus cases being discarded. Based on data from a single Berlin LHD most cases are discarded because of previously diagnosed chronic disease.

Differences in the nature and notification requirements of the diseases under surveillance may explain the range of PPVs across diseases. As physicians are required to report suspected cases of certain diseases (e.g. meningococcal meningitis, measles), it is not surprising that these diseases have generally a lower PPV_CD_ and PPV_RD_ compared to those that are notifiable only by laboratories. The notification of suspected cases is an important aim of the surveillance system as it makes possible that LHDs can take early action (e.g., contact tracing, prophylactic medication or vaccination in case of measles and meningococcal meningitis). Many of these notifications are not true cases at the end, but as the diseases concerned are usually very serious und require rapid intervention, such use of resources is part of the preventive role of public health surveillance that is also the main aim of the notification law. The low PPV of these diseases demonstrates the sensitivity of the alerting function of the system. However, our data suggest that the highest burden of discarded cases for the LHDs arise not from these diseases, but hepatitis B and C.

Our data shows that the range of PPVs across LHDs is wide. Such a difference may be explained by regional differences within the city of Berlin as the density of hospitals and specialty practices is different among districts. Differences can also arise from the varying interpretation of the case definitions resulting in the transmission of non-cases or non-transmission of true cases. Such varying interpretation of case definitions may be the result of misinterpretation (too complex case definitions or insufficient training in the use of them) [11], and, depending on the available resources at the respective LHD, of insufficient investigation of the case resulting in missing data that is relevant for the case definition. Non-transmission of true cases due to misinterpretation of case definitions and insufficient investigation of cases, consequently, may also result in the underestimation of disease incidence of notifiable diseases.

### Discarded notifications

Most of the discarded notifications were hepatitis B and C notifications. For more than two thirds of them the reason for discarding was previously diagnosed chronic disease where some cases had also been notified earlier to the LHD. For 11% of discarded hepatitis B cases the notification was the initial diagnosis of chronic disease.

A direct comparison of our results with that of other countries is difficult as the way of notification and the use of case definitions at the level of LHDs is specific for Germany. However, the high burden of hepatitis B and C notifications that are not true cases from the point of view of the surveillance system, mainly due to non-differentiation between acute and chronic hepatitis and repeated notifications of chronic hepatitis, has been described in other countries [17, 18].

Discarding notified cases with initial diagnosis of chronic hepatitis B may contribute to the underestimation of disease incidence in Germany. In Germany, cases of acute hepatitis B and C (by the physician) and detection of hepatitis B and C virus in acute infection and first-time detection of hepatitis C virus in chronic hepatitis C (by the laboratory) were notifiable at the federal level at the time of this investigation. We can assume that many chronic hepatitis B cases are notified in Germany as some laboratories use automatic, computer-based notification and do not differentiate between acute or chronic disease. These notified and subsequently discarded cases, that are of relevance for estimating disease burden, do not reach higher levels of the public health system as LHDs sort them out using case definitions. The one discarded case of newly diagnosed chronic hepatitis C (Sheet “Discarded_cases_from_1LHD” in S1 File), is most likely the result of misinterpretation of the case definition as all newly diagnosed hepatitis C cases have to be transmitted by the LHD.

The lower PPV of hepatitis C notifications with increasing age could be explained with the increasing prevalence of chronic hepatitis C with age. Young people are more likely to have an acute infection and be tested for the first time in their life compared to older persons who are more likely to have chronic disease.

### Limitations

Our study has several limitations. Although for Berlin we could include data from most LHDs (67%), our data is based on only one federal state of Germany representing about 4% of the population of Germany. However, across Germany notification requirements are the same for all physicians and laboratories and the same case definitions are used. For this reason large differences between states and/or LHDs are expected to be uncommon. Differences within Germany may exist in regard to urban versus rural areas and the varying compliance with mandatory notification across the federal states (e.g., if physicians do not report suspected cases). It would be useful to have comparison data from other states as such a comparison would allow an internal check of the reliability and reproducibility of the notification system in general.

It is also possible that some of the LHDs do not enter some of their discarded notifications in the electronic system leading to overestimation of the PPV. As notifications not entered are most likely those that did not require a high amount of resources (e.g., repeated notifications), their effect is less relevant for estimating the burden arising from discarded cases. The PPV calculated in our study does not measure workload quantitatively and, therefore, it cannot be analysed in economic terms. Together with information about the investigations and measures related to discarded cases, it allows to estimate the proportion of LHD resources that are used for discarded cases and assess the efficiency of the notification system. Additionally, amendments to the notification law have been implemented since conducting the study, including, among others, a shortened time limit for the transmission of a notification to the regional level and new notifiable diseases) [19]. These changes may also influence the overall and disease specific PPVs.

## Conclusions

LHDs discard many notifications, of which a large number require investigation by the LHD. Differences in the nature and notification requirements of diseases under surveillance may explain the range of PPVs. The low PPV and the high case numbers of hepatitis B and C suggest that a relevant amount of LHD resources are used to investigate notifications that do not lead to public health action and useful surveillance data. On the other hand, our results also suggest that many hepatitis B cases considered relevant for disease burden may have already been notified to LHDs, although not required by the notification law, and thus can be used for estimation of disease incidence and epidemiological analyses. In 2015 an update of the German case and reference definitions has been published that requires that hepatitis B cases without symptoms of acute hepatitis are included in the calculation of diseases incidences (they meet the reference definition) [20]. Additionally, in 2017 the notification requirements were changed so that laboratories have to notify every case of hepatitis B or C they detect regardless of clinical symptoms and course of disease (acute or chronic). These cases are then transmitted further and included in disease incidence calculations. These changes are expected to increase the PPV for hepatitis B and C.

For diseases with low PPV, especially those where many true cases are discarded, further adaption of case definitions and reference definitions may lead to better use of data from already notified cases. Changes in the legal framework concerning notifications may further reduce the burden associated with discarded notifications. These may include notification law changes that require notifiers to provide additional data with the notification that helps differentiate chronic and acute hepatitis B and C cases. Additional instructions and clearer notification forms should also be provided for physicians and laboratories. The implementation of a centralized electronic notification system (in which previously notified cases of chronic diseases can be easily identified) could reduce workload by avoiding duplicate notifications [21].

## Supporting information

S1 File Study dataset. (XLS)
